# Changing Patterns in Place of Cancer Death in England: A Population-Based Study

**DOI:** 10.1371/journal.pmed.1001410

**Published:** 2013-03-26

**Authors:** Wei Gao, Yuen K. Ho, Julia Verne, Myer Glickman, Irene J. Higginson, on behalf of the GUIDE_Care project

**Affiliations:** 1 King's College London, School of Medicine, Cicely Saunders Institute, Department of Palliative Care, Policy and Rehabilitation, London, United Kingdom; 2 South West Public Health Observatory, Bristol, United Kingdom; 3 Life Events & Population Sources Division, Office for National Statistics, Newport, Wales, United Kingdom; Hospice Africa, Uganda

## Abstract

**Background:**

Most patients with cancer prefer to die at home or in a hospice, but hospitals remain the most common place of death (PoD).This study aims to explore the changing time trends of PoD and the associated factors, which are essential for end-of-life care improvement.

**Methods and Findings:**

The study analysed all cancer deaths in England collected by the Office for National Statistics during 1993–2010 (*n* = 2,281,223). Time trends of age- and gender-standardised proportion of deaths in individual PoDs were evaluated using weighted piecewise linear regression. Variables associated with PoD (home or hospice versus hospital) were determined using proportion ratio (PR) derived from the log-binomial regression, adjusting for clustering effects. Hospital remained the most common PoD throughout the study period (48.0%; 95% CI 47.9%–48.0%), followed by home (24.5%; 95% CI 24.4%–24.5%), and hospice (16.4%; 95% CI 16.3%–16.4%). Home and hospice deaths increased since 2005 (0.87%; 95% CI 0.74%–0.99%/year, 0.24%; 95% CI 0.17%–0.32%/year, respectively, *p*<0.001), while hospital deaths declined (−1.20%; 95% CI −1.41 to −0.99/year, *p*<0.001). Patients who died from haematological cancer (PRs 0.46–0.52), who were single, widowed, or divorced (PRs 0.75–0.88), and aged over 75 (PRs 0.81–0.84 for 75–84; 0.66–0.72 for 85+) were less likely to die in home or hospice (*p*<0.001; reference groups: colorectal cancer, married, age 25–54). There was little improvement in patients with lung cancer of dying in home or hospice (PRs 0.87–0.88). Marital status became the second most important factor associated with PoD, after cancer type. Patients from less deprived areas (higher quintile of the deprivation index) were more likely to die at home or in a hospice than those from more deprived areas (lower quintile of the deprivation index; PRs 1.02–1.12). The analysis is limited by a lack of data on individual patients' preferences for PoD or a clinical indication of the most appropriate PoD.

**Conclusions:**

More efforts are needed to reduce hospital deaths. Health care facilities should be improved and enhanced to support the increased home and hospice deaths. People who are single, widowed, or divorced should be a focus for end-of-life care improvement, along with known at risk groups such as haematological cancer, lung cancer, older age, and deprivation.

*Please see later in the article for the Editors' Summary*

## Abstract 2

Wei Gao and colleagues describe how location of death has changed for patients with cancer in England between 1993 and 2010.

## Editors' Summary

**Background:**

Death is the only certain event in our lives. Consequently, end-of-life care is an issue that is relevant to everyone, and everyone hopes for a “good death” (a death that is free from avoidable distress and suffering) for themselves and for their loved ones. Many factors contribute to a good death, including the place of death. When asked, most people say they would rather die at home or in a hospice (a care facility that gives supportive care to people in the final phase of a terminal illness and that focuses on comfort and quality of life rather than on cure) than in a hospital. Importantly, patients who die at home or in a hospice often have a better quality of life than those who die in hospital, and caring for terminally ill patients in the community is less expensive than caring for them in hospital.

**Why Was This Study Done?:**

Many countries have introduced end-of-life care policies that are designed to enable more people to die at home or in hospices. England, for example, implemented its National End of Life Care Programme in 2004. However, to improve end-of-life care services and to enable more people to die in their preferred place, we need to understand how the patterns of place of death and the factors that affect the place of death are changing over time. In this population-based study, the researchers examine the changing pattern of place of death of people with cancer and the factors associated with place of death in England between 1993 and 2010. Cancer is a leading cause of death globally and is responsible for 8 million deaths annually. Deaths from cancer still occur most commonly in hospitals, which are the least preferred place of death for people with cancer; home and hospices are the first and second preferred places of death, respectively, for such people.

**What Did the Researchers Do and Find?:**

The researchers used death registration data collected by the Office of National Statistics to identify all the adult cancer deaths in England between 1993 and 2010 (2.28 million deaths) and to determine where these deaths occurred, time trends in place of death, and the factors associated with place of death. Hospital was the commonest place of death throughout the study period—48% of cancer deaths occurred in hospital, 24.5% at home, and 16.4% in hospices. The proportion of home deaths increased after 2005 whereas the proportion of hospital deaths declined. The proportion of deaths in hospices also increased over the study period. The most important factor associated with place of death was cancer site. For example, patients who died from a blood (hematological) cancer were more likely to die in hospital than patients with colorectal cancer throughout the study period although the proportion dying at home or in a hospice increased over time. Being single, widowed, or divorced was associated with a higher likelihood of dying in hospital than being married. Being over 75 was associated with a higher likelihood of dying in hospital than being 25–54 although elderly people were more likely to die at home or in a hospice after 2006 than in earlier periods.

**What Do These Findings Mean?:**

These findings show that the hospital is still the commonest place of death for patients with cancer in England. However, the increase in home and hospice deaths since 2005 suggests that the National End of Life Care Programme has enabled more people to die at their preferred place of death. These findings identify cancer site, marital status, and age as the three most important factors associated with place of death for patients with cancer. Because the study is a large-scale, population-based study, these findings are likely to be generalizable to other high-income settings. However, because the study did not include data on individual patient preferences for place of death, these findings should be applied with care to individual patients. Importantly, these findings indicate that more needs to be done to support people with cancer (and other terminal illnesses) who wish to die at home or in a hospice. Moreover, they identify groups of people—single, widowed or divorced individuals, older people, and people with specific types of cancer—who need extra help to ensure that they are able to choose where they die.

**Additional Information:**

Please access these Web sites via the online version of this summary at http://dx.doi.org/10.1371/journal.pmed.1001410.

- The Cicely Saunders International, a not-for-profit organization, promotes research to improve the care and treatment of all patients with terminal illnesses at home, in hospices and in hospital; its website includes information on end-of-life care and on Cicely Saunders, the founder of the hospice movement in England
- This study is part of GUIDE_Care, a project initiated by the Cicely Saunders Institute to investigate patterns in place of death and the factors that affect these patterns
- Information on the National End of Life Care Programme is available
- The UK National Health Service Choices website provides information (including videos of personal experiences) on end-of-life issues for carers, information on end-of-life care for patients with cancer, and an end-of-life care guide for people approaching the end of their life
- The US National Cancer Institute has a fact sheet on end-of-life care for people who have cancer and provides information on hospice care and home care for patients with cancer (in English and Spanish)
- The not-for-profit organization HealthTalkOnline provides personal stories about living with dying
- The NHS National End of Life Intelligence Network (NEoLCIN) provides information on broad issues about end-of-life care
- The South West Public Health Observatory (SWPHO) aims to improve the health of the population through producing evidence to inform decision making on health issues at local, regional, and national levels. SWPHO also produces specific end-of-life care resources (e.g., specialised reports, end-of-life care profiles) and disseminates it via the NEoLCIN website

## Introduction

End-of-life care is an issue that is relevant to everyone, as death is the only certain factor of life. Every year, around 8 million people die of cancer worldwide and the global number of cancer deaths is projected to increase [1]. Patients with cancer usually prefer to die at home or a hospice should they have a choice, particularly in high resource European countries [2]. However, cancer deaths still most commonly occur in hospitals, which is consistently regarded as the least preferred place of death (PoD) [2]. Patients with cancer who die in a hospital or intensive care unit (ICU) have worse quality of life compared with those who die at home, and their bereaved caregivers are at increased risk for developing psychiatric illness [3]–[5]. Meeting people's preferences for PoD also has cost implications [6]. Research found that end-of-life care in hospital is associated with three times higher daily costs than in community care settings [7]. Therefore, reducing inappropriate deaths in hospital, and increasing home and hospice support, has become a central focus of policy initiatives in many countries around the world [8]–[14].

Over the past decade, resources have been directed to enable more people, mainly those with cancer, to die in their preferred place. In England since the 1990s, several national end-of-life care initiatives have been established and implemented [8]. In 2004, a National Health Service (NHS) End of Life Care (EoLC) programme was further established to promote the rollout of national end-of-life care initiatives [15]. Although valuable for policy review and oversight, to our knowledge no study has evaluated the time trend of place of cancer deaths in the context of these programmes. For the development of effective intervention strategies and end-of-life care policies, it is essential to understand the factors associated with PoD in a dynamic rather than a static way. Understanding patterns of PoD in England has value for other countries. The health care system in England is provided and financed by the government through taxation (Beveridge), and represents one of four health care models (Beveridge, Bismarck, national health insurance, out-of-pocket) worldwide [13]. Furthermore, the modern hospice movement, which offers assistance to patients with advanced diseases and their families, was started by Dame Cicely Saunders in England in 1967, and has spread globally. In England, most hospice care is provided by charitable hospices with a contribution from the NHS; it is free and provided on the basis of physical, psycho-social, and spiritual needs. Consistent evidence shows that hospice is usually considered as the second most preferred PoD, next to home; however, not all countries have the similar level of hospice service provision or models [16]. England's evaluation data on PoD may provide useful insights for end-of-life care service development with different countries and health care settings.

Patterns in place of cancer deaths in England were investigated using death registry data for 1984–1994, but there has been no subsequent in-depth update [17]. One study using data from a regional cancer registry found PoD for people with cancer changed over time; but it was restricted to only the population covered by the registry, limiting the generalisability and applicability of the findings [18]. To make effective improvements in end-of-life care services and enable more people to die in their preferred place, it is essential to understand the factors associated with PoD and how those factors change over time. To our knowledge, no study has evaluated this aspect, or considered the relationship between possible factors and PoD in the context of change. In the UK and worldwide, the national Death Registration database has been used as an important tool for public health planning and surveillance [1],[12] [13]. It has been proposed as an ideal population-based data source for end-of-life care research [19].

This study aimed to investigate the changing patterns in the: (i) common places of cancer death; (ii) time trends in place of cancer death, and (iii) factors associated with place of cancer death and their relative importance.

## Methods

### Data Sources

Data are collected by the Office for National Statistics (ONS) from all death registrations in England. By law in England, a death must be registered within 5 d, unless it becomes the subject of a coroner's inquiry. The underlying cause of death (CoD) was recorded in the database using the 9th (1993–2000) or 10th (2001–2010) edition of International Classification of Diseases (ICD-9, ICD-10) codes.

### Study Population

All deaths between 1993 and 2010 where cancer was the underlying CoD (ICD-10: C00–C97; ICD-9: 140–209) were extracted. We limited the analysis period to 1993 onwards, since hospice was only recorded as a separate PoD category from 1993. As there are important differences between children/young and older people in terms of disease profile, end-of-life care model, and associated infrastructure (e.g., hospice provision), we focused on those who died aged over 25. Any benign, in-situ neoplasms and neoplasms of unknown and uncertain behaviour were excluded as the accuracy of these underlying CoD is questionable [20].

### Variables

The PoD was grouped into five categories: home, hospital, hospice, other communal establishments (including nursing home, residential home, and care home), and elsewhere. Hospice refers to a dedicated unit with in-patient beds. These are usually freestanding from hospitals. Explanatory variables included: age at death (25–54, 55–64, 65–74, 75–84, 85+), gender (male, female), cancer site (See Table 1 for ICD-9/10 codes), year of death, marital status (single, divorced, widowed, married, not stated/unknown), the socio-economic status (SES), and region (defined by Strategic Health Authorities [SHAs], 2006). We analysed age as an ordered five-category rather than a continuous variable to aid interpretation and comparison with the other studies; the cut-off boundaries were chosen based on the data distribution [17],[21],[22]. The SES was measured by the quintile of a deprivation index, index of multiple deprivation (IMD: 1, most deprived; 5, least deprived), that was commonly used in the corresponding periods: IMD 2000 for 1993–2000, IMD 2010 for 2001–2010 [23]. The IMD is an area-specific deprivation measure for small geographical areas (lower layer super output areas [LSOAs]) in England. It is a weighted average score of seven distinct domains, including income; employment; health and disability; education, skills, and training deprivation; barriers to housing and services; living environment; and crime deprivation. A LSOA is a low-level geographic area that is designed for reporting small area statistics in England and Wales. There are 32,482 LSOAs in England; each area has a minimum population size of 1,000 and an average of 1,500. LSOAs were allocated into quintile deprivation categories on the basis of their IMD scores [23].

**Table 1:** ICD-9 and ICD-10 codes used for the classification of underlying cause of death as cancers.

Cancer	ICD-9 Codes (1993–2000)	ICD-10 Codes (2001–2010)
Bladder	188	C67
Breast	174	C50
Colorectal	153,154	C18–C20
Head and neck	141,143–148,161	C00–C14,C30–C32
Kidney	189	C64–C66, C68
Haematology	200–209	C81–C96
Liver	155	C22
Lung	162	C33–C34
Oesophagus	150	C15
Ovarian	183	C56–C57
Pancreas	157	C25
Prostate	185	C61
Stomach	151	C16
Others	140–209excluding above codes	C00–C97excluding above codes

### Statistical Analysis

We analysed the time trend of age- and gender-standardised proportion of deaths in each of the five PoDs. Proportions were standardised using the 2005–2010 mortality structure for more developed countries from the United Nations standard population [24]. Age- and gender-adjusted proportions were plotted against year of death. Changes in proportions were inspected visually. Then we conducted a weighted piecewise linear regression (WPLR) analysis to confirm the findings [25]. The WPLR model was run separately for the five PoDs. In these analyses, the time trend was analysed across the whole period (1993–2010) and year of death was evaluated as a continuous variable.

To examine changing patterns, the study period was divided into four: 1993–1995, 1996–2000, 2001–2005, 2006–2010. The division took into consideration major changes in coding schemes, including PoD and ICD coding system. These included: (i) “Hospice” was coded as a separate category from 1993; (ii) the ICD coding system changed from the ICD-9 to the ICD-10 in 2001.

The log-binomial model was used to investigate factors associated with PoD. The dependent variable was a binary indicator for PoD (1, home or hospice; 0, hospital). In these analyses, we focused on the top three PoDs. “Home” and “hospice” categories were combined because these are usually the two most preferred PoDs, whereas “hospital” is the least preferred [2],[26]. The clustering effect within the geographical units (LSOA) was adjusted using the general estimating equation (GEE) method, assuming an exchangeable working correlation matrix. Explanatory variables (age, gender, cancer site, year of death, marital status, SES, and regions) were forced to stay in the model. The relative importance of explanatory variables was determined by the change in the score statistics between the full and the reduced models [27]. The PRs for individual explanatory variables were derived from the constructed period-specific models. Two-way interaction effects between important factors were explored. All analyses were performed using the SAS 9.3 (SAS Institute).

### Ethics and Permission

Following ONS procedures a Data Access Agreement was signed and all required forms provided in a formal agreement of data management, protection, and management. In addition, as required, all researchers accessing the data (WG, IJH, and HK) were individually assessed and approved by ONS. This study was based on fully anonymised records therefore no ethical approval was required according to the Information Commissioner's Office (ICO) guidelines, ONS procedures, and those of the King's College London Research Ethics Committee.

## Results

After exclusion of 3,968 (0.2%) delayed registrations and 23,746 (1.0%) with missing data on key variables, the final data set consisted of 2,281,223 adults (≥25 y) who died from cancer between the years 1993–2010 in England. The annual deaths fluctuated between 121,197 and 129,941.

Patients with cancer died increasingly at older ages; the proportion of cancer patients who died over age 65 rose from 43.7% in 1993–1996 to 52.0% in 2006–2010 (Table 2). Slightly more men than women died of cancer over the whole study period (52.2% versus 47.8%). The three most common causes of cancer death were lung (22.1%), colorectal (10.6%), and breast (8.5%). There was a slight rise in deaths from haematological cancers (6.9%–7.2% in 1993–2000 to 7.7 in 2001–2010). Nearly half (47.0%) of people that died were single, widowed, or divorced. While cancer deaths were more likely to be residents from deprived areas in earlier periods (e.g.,14.3%–14.7% least deprived versus 29.9%–30.9% most deprived in 1993–2000), they became more evenly distributed across socio-economic groups in the later periods (17.8%–18.6% least deprived versus 20.1%–21.1% most deprived in 2001–2010). The North West and North East area represented the lowest (6.1%) and the highest (14.9%) percentage of cancer deaths, approximately reflecting the underlying population size of the regions.

**Table 2:** Demographic characteristics of all deaths with cancer as the underlying cause of death in England, 1993–2010.

Characteristic	Subgroup	Year of Death				All
		1993–1995	1996–2000	2001–2005	2006–2010	
All	Total deaths	388,176	625,037	630,952	637,058	2,281,223
All	Average annual deaths	129,392	125,007	126,190	127,412	126,735
Age	25–54	9.6	9.5	8.5	7.8	8.8
	55–64	15.1	14.6	14.9	15.1	14.9
	65–74	31.7	29.2	26.3	25.1	27.7
	75–84	30.6	32.0	33.9	33.0	32.6
	85+	13.1	14.7	16.4	19.0	16.1
Gender	Male	52.4	52.1	52.0	52.4	52.2
	Female	47.6	47.9	48.0	47.6	47.8
Cancer site	Bladder	3.5	3.4	3.2	3.3	3.3
	Breast	9.3	8.8	8.4	7.8	8.5
	Colorectal	11.5	11.1	10.3	10.1	10.6
	Haematologic	6.9	7.2	7.7	7.7	7.5
	Head and neck	1.5	1.5	1.9	1.9	1.7
	Kidney	1.9	2.0	2.2	2.5	2.2
	Liver	1.2	1.4	1.7	2.2	1.7
	Lung	23.3	22.4	21.2	21.8	22.1
	Oesophagus	4.1	4.4	4.7	4.8	4.6
	Other	18.2	19.2	20.6	20.0	19.6
	Ovarian	2.8	3.0	3.0	2.8	2.9
	Pancreas	4.2	4.4	4.6	5.1	4.6
	Prostate	6.3	6.4	6.7	6.8	6.6
	Stomach	5.3	4.7	3.9	3.3	4.2
Marital status	Married	53.4	52.9	52.0	51.7	52.4
	Widowed	32.0	32.2	31.9	30.5	31.6
	Single	8.3	8.1	7.9	7.8	8.0
	Divorced	5.3	6.3	7.8	9.4	7.4
	Not stated/unknown	1.0	0.5	0.5	0.6	0.6
IMD	1 (most deprived)	30.9	29.9	21.1	20.1	24.9
	2	22.2	22.2	20.4	19.9	21.1
	3	17.8	18.1	20.7	20.8	19.5
	4	14.8	15.1	20.0	20.6	17.9
	5 (least deprived)	14.3	14.7	17.8	18.6	16.6
Region^a^	North East	6.1	6.1	6.0	6.0	6.1
	North West	15.0	15.1	14.9	14.7	14.9
	Yorkshire and the Humber	10.6	10.6	10.6	10.6	10.6
	East Midlands	8.3	8.4	8.6	8.8	8.6
	West Midlands	10.8	10.8	10.8	10.9	10.8
	East of England	10.3	10.5	10.9	11.2	10.8
	London	12.5	12.0	11.3	10.6	11.5
	South East Coast	9.1	8.9	8.9	8.9	9.0
	South Central	6.7	6.9	7.0	7.2	7.0
	South West	10.5	10.7	11.0	11.2	10.9
PoD	Hospital	49.0	48.5	49.9	44.9	48.0
	Home	26.2	24.0	22.4	25.8	24.5
	Hospice	13.6	16.6	16.8	17.4	16.4
	Other communal establishments	9.6	9.4	9.6	10.6	9.8
	Elsewhere	1.6	1.6	1.3	1.2	1.4

a The region was defined by Strategic Health Authorities (SHAs) (July 2006).

**Figure 1:**
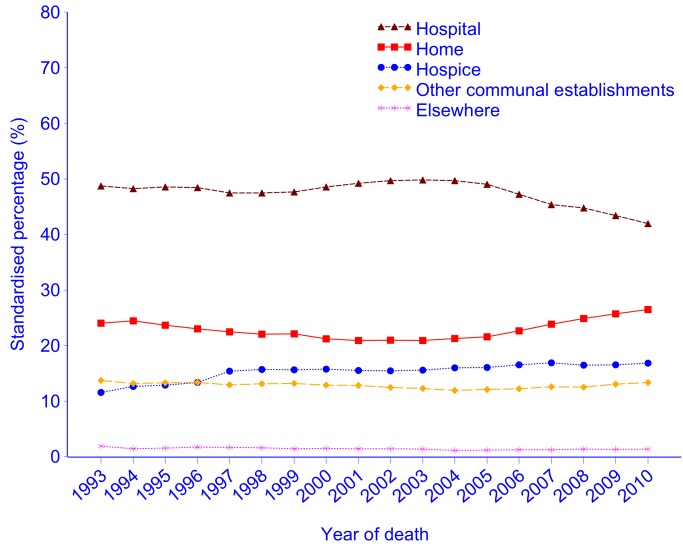
Place of cancer deaths in England, 1993–2010, age- and gender-standardised against the UN mortality standard population [**24**]. The 95% CIs were not plotted as they were too narrow to show.

Throughout the study period, hospital was the most common PoD (48.0%; 95% CI 47.9%–48.0%), followed by home (24.5%; 95% CI 24.4%–24.5%), and hospice (16.4%; 95% CI 16.3%–16.4%). Age- and gender-adjusted deaths at home had a slight but constant downward trend (annual reduction: −0.38% [95% CI −0.45% to −0.31%]; *p*<0.001) between 1993 (24.0%; 95% CI 23.6%–24.4%) and 2003 (20.9%; 95% CI 20.5%–21.3%); however, this trend started reversing in 2003/2004 and the proportion of home deaths rose to 26.5% (95% CI 26.1%–26.9%) in 2010 (annual increase: 0.87%; 95% CI 0.74%–0.99%; *p*<0.001). The weighted piecewise linear regression (WPLR) analysis identified a significant increase in home deaths in 2005/2006 (21.6%; 95% CI 21.2%–21.9%) to 22.7% (95% CI 22.3%–23.1%; *p*<0.001, *F*_df = 1_ = 25.5). The hospital deaths fluctuated between 47.5%%–49.8% before a significant reduction of 1.82% (95% CI 1.81%–1.83%; *p*<0.001, *F*_df = 1_ = 152.9) in 2005/2006, mirroring the change in home deaths (annual increase: 0.87%; 95% CI 0.74%–0.99%). Hospital deaths continued to decline after then, to 41.9% (95% CI 41.4%–42.5%) in 2010 (annual decrease: −1.20%; 95% CI −1.41 to −0.99%; *p*<0.001). Deaths in hospice showed an overall increasing trend (annual increase: 0.24%; 95% CI 0.17%–0.32%; *p*<0.001), from 11.6% (95% CI 11.3%–11.8%) in 1993 to 16.8% (95% CI 16.5%–17.2%) in 2010. A 2.04% (95%CI 2.02%–2.06%) increase in hospice deaths was detected in 1996/1997 (*p*<0.001, *F*_df = 1_ = 84.3) (Figure 1).

The relative importance of factors associated with where a cancer patient died (Table 3) changed over time. Cancer site was the most important factor associated with PoD (score statistics range: 2257.0–5835.4; *p*<0.001) in all time periods. Marital status was the second most important factor during 1993–1995 (1,602.8 versus 1,471.6), but became the third during 1996–2005 (2,218.7 versus 2,321.9 in 1996–2000; 3,409.0 versus 3,983.9 in 2001–2005); in 2006–2010, it was again second (3,369.8 versus 3,297.0).

**Table 3:** Proportion ratios and 95% CIs of variables associated with place of death (home/hospice versus hospital) in England 1993–2010.

Variable	Value	1993–1995		1996–2000		2001–2005		2006–2010	
		PR	95% CI	PR	95% CI	PR	95% CI	PR	95% CI
Age	25–54	1.00	—	1.00	—	1.00	—	1.00	—
	55–64	0.99	0.98–1.00	0.97	0.96–0.98	0.96	0.95–0.97	0.95	0.94–0.96
	65–74	0.94	0.93–0.95	0.93	0.92–0.94	0.91	0.90–0.92	0.91	0.90–0.92
	75–84	0.83	0.82–0.85	0.83	0.82–0.84	0.81	0.80–0.82	0.84	0.83–0.85
	85+	0.70	0.69–0.71	0.67	0.66–0.68	0.66	0.65–0.67	0.72	0.71–0.72
Gender	Female	1.00	—	1.00	—	1.00	—	1.00	—
	Male	0.97	0.96–0.97	0.96	0.95–0.97	0.95	0.94–0.95	0.96	0.95–0.96
Cancer	Colorectal	1.00	—	1.00	—	1.00	—	1.00	—
	Bladder	0.78	0.77–0.80	0.83	0.82–0.85	0.82	0.81–0.84	0.84	0.82–0.85
	Breast	0.89	0.88–0.91	0.91	0.90–0.92	0.89	0.88–0.90	0.91	0.90–0.92
	Haematology	0.46	0.45–0.47	0.48	0.47–0.49	0.47	0.46–0.48	0.52	0.51–0.53
	Head and neck	0.97	0.94–0.99	0.97	0.94–0.99	0.96	0.94–0.98	0.97	0.95–0.99
	Kidney	0.92	0.90–0.94	0.98	0.96–0.99	0.98	0.96–1.00	0.96	0.95–0.98
	Liver	0.82	0.79–0.84	0.84	0.82–0.86	0.83	0.81–0.85	0.87	0.86–0.89
	Lung	0.88	0.87–0.89	0.87	0.86–0.88	0.87	0.86–0.88	0.88	0.87–0.89
	Oesophagus	0.90	0.88–0.92	0.93	0.92–0.94	0.94	0.92–0.95	0.96	0.95–0.97
	Ovarian	0.90	0.88–0.92	0.92	0.91–0.94	0.91	0.89–0.92	0.95	0.93–0.96
	Pancreas	0.93	0.92–0.95	0.97	0.96–0.99	0.96	0.95–0.98	0.99	0.98–1.00
	Prostate	0.87	0.86–0.89	0.95	0.94–0.96	0.94	0.92–0.95	0.93	0.92–0.95
	Stomach	1.01	0.99–1.03	1.02	1.01–1.04	1.03	1.02–1.05	1.04	1.03–1.06
	Other	0.81	0.80–0.82	0.83	0.82–0.84	0.82	0.82–0.83	0.88	0.87–0.89
Marital status	Married	1.00	—	1.00	—	1.00	—	1.00	—
	Divorced	0.86	0.85–0.88	0.86	0.85–0.87	0.86	0.85–0.87	0.88	0.87–0.89
	Single	0.75	0.74–0.77	0.75	0.74–0.76	0.75	0.74–0.76	0.76	0.75–0.77
	Widowed	0.84	0.83–0.85	0.84	0.83–0.84	0.83	0.82–0.84	0.86	0.85–0.86
	Not stated/unknown	0.91	0.88–0.95	0.88	0.84–0.92	0.82	0.78–0.86	0.83	0.80–0.86
SES	1 Most deprived	1.00	—	1.00	—	1.00	—	1.00	—
	2	1.03	1.01–1.04	1.03	1.01–1.04	1.03	1.02–1.05	1.02	1.01–1.04
	3	1.04	1.02–1.06	1.04	1.03–1.06	1.06	1.05–1.08	1.06	1.05–1.07
	4	1.05	1.03–1.07	1.06	1.04–1.07	1.10	1.09–1.12	1.09	1.08–1.11
	5 Least deprived	1.06	1.04–1.08	1.07	1.05–1.08	1.12	1.11–1.13	1.11	1.10–1.13
Region	North West	1.00	—	1.00	—	1.00	—	1.00	—
	East England	0.88	0.86–0.90	0.92	0.90–0.94	0.93	0.91–0.94	0.95	0.94–0.96
	East Midlands	0.80	0.78–0.82	0.85	0.84–0.87	0.86	0.85–0.87	0.88	0.86–0.89
	London	0.81	0.79–0.83	0.82	0.80–0.84	0.87	0.86–0.89	0.89	0.87–0.90
	North East	0.85	0.83–0.88	0.91	0.89–0.93	0.91	0.89–0.92	0.88	0.87–0.90
	South Central	0.90	0.87–0.92	0.98	0.95–1.00	0.99	0.97–1.01	0.97	0.95–0.98
	South East Coast	1.03	1.01–1.06	1.02	1.00–1.05	1.05	1.04–1.07	1.06	1.05–1.08
	South West	0.94	0.92–0.97	0.92	0.90–0.94	0.93	0.92–0.95	0.98	0.97–1.00
	West Midlands	0.97	0.95–0.99	0.95	0.93–0.96	0.92	0.91–0.93	0.95	0.94–0.96
	Yorkshire and Humber	0.95	0.93–0.98	0.93	0.91–0.95	0.97	0.96–0.98	0.96	0.95–0.97

PRs were estimated from the log-binomial regression models. The clustering effect within the LSOA geographical units was adjusted using the general estimating equation (GEE) method. In additional to variables listed in the table, models were also adjusted for the calendar year of death. A PR greater than 1 indicates higher probability of death at home/hospice than the reference category. The *p*-value for overall association of individual factors with PoD was smaller than 0.001 in all models.

Overall, the gaps in PoD between subgroups of the three most important factors (cancer, marital status, and age; reference group: colorectal cancer, married and age 25–54) narrowed over time; this became more pronounced in 2006–2010 (Table 3). People with haematological cancers had the lowest chance of dying in home or hospice, but their likelihood of home or hospice death increased over time: the PR rose from 0.46 (95% CI 0.45–0.47) in 1993–1995 to 0.52 (95% CI 0.51–0.53) in 2006–2010. There was little improvement in chances of dying in home or hospice in lung cancer (PRs: 0.87–0.88), the most common cancer group. People who died aged over 85 were more likely to die in home or hospice in 2006–2010 (PRs: 0.72, 95% CI 0.71–0.72), than in earlier periods (PRs: 0.66–0.70). The inequality in home/hospice death by marital status slightly improved: the PRs for all status other than “married” in 2006–2010 were the highest (though marginal) among subperiods (single:0.76, 95% CI 0.75–0.77; widowed:0.86, 95% CI 0.85–0.86; divorced:0.88, 95% CI 0.87–0.89).

The gaps between subgroups for less important factors (gender, SES, and region) remained stable. Men were less likely to die at home or in a hospice (PRs versus women: 0.95–0.97); patients from less deprived areas were more likely to die at home or in a hospice than those from more deprived areas (PRs:1.02–1.12). The PR difference between regions with the lowest (East Midlands or London) and highest (South East Coast) chance of death in home or hospice in four periods were: 0.23 (1993–1995), 0.20 (1996–2000), 0.19 (2001–2005), 0.18 (2006–2010). London was the lowest in 1993–2000, but was replaced by East Midlands from 2001. We also tested all models with age included as a continuous variable, the parameter estimates (Table S1, S2, S3) remained very similar to those in Table 3.

The two-way interaction effect between the top three most important factors were all significant at the level of *p*<0.0001, but there was no substantial change in the parameter estimates for main effects by including the interaction term in the models (Table S4).

## Discussion

This large-scale, population-based study found that hospitals remain the most common PoD for patients with cancer. Following a prolonged period of plateau, there was a steady downward trend in hospital deaths (to about 50%) from 2005 onwards. The pattern was mirrored by increasing home deaths; this was confirmed by statistical modelling. This trend coincides with the launch of a National End of Life Care (EoLC) Programme in Nov 2004 in England [15], which was based on research evidence about patient preferences and possible solutions. This trend continued steadily since 2005 [16],[17],[26]. The programme aimed to reduce hospital deaths and enable more people to die at a place of their choice, usually own home or a hospice, through promoting good practice in end-of-life care. Compared to people with other diseases, patients with cancer had better access to end-of-life care facilities [8],[28]. One would expect that patients with cancer would be the first beneficiaries of national initiatives on end-of-life care. An earlier report on PoD using aggregated level data in England and Wales found a similar but less pronounced reversal trend for all cause home deaths [22]. Findings from this cancer focused study, together with earlier research on all-cause deaths, provide some support for the effectiveness of national initiatives in improving end-of-life care; however, this needs to be confirmed by subsequent studies.

There was a tendency for the inequality gap in PoD to become smaller; however, little improvement exists for those with certain types of cancer. For example, in 2006–2010, deaths from haematological cancer were still 50% more likely to have occurred in hospitals compared with colorectal cancer. These results were consistent with other studies [9],[18],[29]–[31]. Reasons for high hospital deaths in haematological cancer may be related to the complex care transition inherent to haematological malignancies, disease symptoms, the side effects of chemotherapy, or limited links between haematology and palliative care services [30]. However, current evidence is fragmented and derived from studies with limited generalisability. Future studies are needed to examine the risk factors for hospital death in heamatological malignancies. Lung cancer, one of the most common cancers in the UK and worldwide [1], showed little improvement in the chances of dying in home or hospice. A recent cohort study on 2,155 patients with advanced cancer suggests that this outcome might be related to late discussions of end-of-life care planning [32].

We also found that marital status is becoming increasingly important as a risk factor for hospital death. In 2006–2010, marital status became the second most important determinant for PoD. Married patients with cancer had significantly better chances of dying in home or hospice than their single, divorced, or widowed peers. Our data support findings in other countries [33]–[35]. Married individuals may receive more home support from their partners, enabling them more likely to die in their preferred place [31]. Individuals from more deprived areas had higher hospital deaths, perhaps suggesting that social support may play an important role in PoD. However, there is little evidence on the causes of this inequality, which might also include accessibility of out-of-hours care services, limited knowledge of available care options, or inability to bear the costs of caring at home. Policy-makers and health services planners should take into consideration the support from partners or spouses, other family members, and the local community in order to design effective end-of-life care services.

In the current era of an aging population, it is particularly important to keep standards of end-of-life care at the highest level for older people. Substandard health care generally is more frequently reported in older rather than in younger people [36],[37]. This inequity exists also in the treatment of cancer pain. Compared with those under 50 y, patients with cancer aged over 80 y are less than half as likely to be prescribed appropriate strong analgesics [38]. Inadequate symptom management may be a reason for hospital admission. We found that age was negatively associated with the likelihood of death at own home or a hospice, but the gap between young and old is getting smaller. This is an encouraging trend, if home is the preferred PoD and quality is high. A preference is influenced by prior experience, positive or negative, and patients may change preference if they have a poor experience of home or hospital care [26]. Some older people also fear to be a burden to relatives or friends [39]. Population-based studies of preferences have shown that the majority of older people do want to remain at home or (for those aged over 85 y) in a hospice [2]. Therefore, research is needed on ways to improve the quality of home care for older people, to meet preferences and ensure symptom management and quality.

A worrying trend is emerging; the gaps in PoD between those who lived in advantaged and disadvantaged areas widened in recent periods (2001–2010), in contrast to converging trends for the other factors. This may be prone to ecological fallacy, as the deprivation was measured at the area level. It needs to be further investigated, ideally, measuring deprivation at the individual level. Nevertheless, it suggests that the End of Life Care (EoLC) strategy may not yet be influencing the poorest communities in England, and action should be targeted to these areas. We also observed significant variations in PoD across regions; the south coast area has the highest chance of death occurring in home or hospice. Although the difference between the regions has become less variable over time, it persists; even taking into account all available and seemingly important confounders. In-depth comparative studies may help to reveal the crucial factors that drive these differences.

### Strengths and Limitations

The strength of this study is its large-scale population-based design. The findings can be directly applied to inform national policies, identify gaps and assess needs, and allocate resources on the end-of-life care; they can also be applied to the countries with similar health care models. The study provided high quality empirical evidence to support findings from a previous systematic review on factors influencing the PoD [31]. In addition, we identified older age as one of the most important factors that had not been highlighted in that review, although international evidence was not consistent about age being a risk factor [31]. We were able to investigate the changing patterns in PoD and its associated factors over a long period of time (18 y). We used PR rather than odds ratio (OR) to estimate relative risk. The OR has been criticised because it may overestimate the true relative risk when applied to common outcomes, which is the case with most PoD studies. However, we can only adjust for a limited number of factors that are potentially associated with PoD. For variables measured at the area level (e.g., SES) there might be risk of committing ecological fallacy. For example, patients living in a specific area may not necessarily have the same SES with that area. We did not have data on individual patients' preferences for PoD or a clinical indication of the most appropriate PoD. But at the population level, hospital has been consistently considered as the least preferred PoD, independent of many factors such as country, setting, or diagnosis [2],[14],[26],[40]. Hence, to reduce hospital death has become a major target of the public health policies for end-of-life care [12],[13],[15]. Nevertheless, caution should be exercised when applying our findings to individual patients.

In conclusion, hospital remained the most common PoD for patients with cancer in England (48%). Home and hospice deaths increased since 2005, oppositely mirroring reducing trends in hospital deaths. People who died from haematological cancer, who were single, widowed or divorced, and aged over 75 y, were less likely to die in home or hospice. There was little improvement in patients with lung cancer of dying in home or hospice. Marital status overtook age as the second most important factor associated with PoD, after cancer type. More efforts are needed to reduce hospital deaths. Health care facilities should be improved and enhanced to support the increased home and hospice deaths. People who are single, widowed, or divorced should be a focus for end-of-life care improvement, along with known at risk groups such as haematological cancer, lung cancer, older age, and deprivation.

## Supporting Information

Table S1 **Proportion ratios and 95% CIs of variables associated with place of death (home/hospice versus hospital) in England 1993–2010; with the interaction term of age and cancer and showing only main effects.**
(DOCX)

Table S2 **Proportion ratios and 95% CIs of variables associated with place of death(home/hospice versus hospital) in England 1993–2010; with the interaction term of age and marital status and showing only main effects.**
(DOCX)

Table S3 **Proportion ratios and 95% CIs of variables associated with place of death(home/hospice versus hospital) in England 1993–2010; with interaction term of cancer and marital status and showing only main effects.**
(DOCX)

Table S4 **Proportion ratios and 95% CIs of variables associated with place of death (home/hospice versus hospital) in England 1993–2010; age at death was modelled as a continuous variable.**
(DOCX)

## Abbreviations

IMD: index of multiple deprivation
LSOA: layer super output area
ONS: Office for National Statistics
PoD: place of death
PR: proportion ratio
SES: socio-economic status
