# Supplementary material for: Changing Patterns in Place of Cancer Death in England: A Population-Based Study
Source: PLoS Med. 2013 Mar 26;10(3):e1001410. doi: 10.1371/journal.pmed.1001410 (PMC3608543; doi:10.1371/journal.pmed.1001410)
Supplement: Table S1 — Proportion ratios and 95% CIs of variables associated with place of death (home/hospice versus hospital) in England 1993–2010; with the interaction term of age and cancer and showing only main effects. (DOCX) [file pmed.1001410.s001.docx]

**Table S1. Proportion ratios and 95% CIs of variables associated with place of death(home/hospice vs hospital) in England 1993-2010; with the interaction term of age and cancer and showing only main effects**

| Variable | Value | 1993-1995 | | 1996-2000 | | 2001-2005 | | 2006-2010 | |
| --- | --- | --- | --- | --- | --- | --- | --- | --- | --- |
|  |  | PR | 95%CI | PR | 95%CI | PR | 95%CI | PR | 95%CI |
| Age | 25-54 | 1.00 | -- | 1.00 | -- | 1.00 | -- | 1.00 | -- |
|  | 55-64 | 0.98 | 0.95 ,1.01 | 0.98 | 0.95 ,1.01 | 0.99 | 0.96 ,1.02 | 0.96 | 0.93 ,0.99 |
|  | 65-74 | 0.93 | 0.90 ,0.96 | 0.91 | 0.89 ,0.93 | 0.91 | 0.88 ,0.93 | 0.90 | 0.88 ,0.92 |
|  | 75-84 | 0.78 | 0.75 ,0.80 | 0.78 | 0.76 ,0.80 | 0.78 | 0.76 ,0.80 | 0.83 | 0.81 ,0.85 |
|  | 85+ | 0.63 | 0.60 ,0.66 | 0.61 | 0.59 ,0.63 | 0.63 | 0.61 ,0.65 | 0.71 | 0.69 ,0.74 |
| Gender | Female | 1.00 | -- | 1.00 | -- | 1.00 | -- | 1.00 | -- |
|  | Male | 0.97 | 0.96 ,0.97 | 0.96 | 0.95 ,0.96 | 0.95 | 0.94 ,0.96 | 0.96 | 0.95 ,0.96 |
| Cancer | Colorectal | 1.00 | -- | 1.00 | -- | 1.00 | -- | 1.00 | -- |
|  | Bladder | 0.78 | 0.70 ,0.87 | 0.88 | 0.82 ,0.95 | 0.91 | 0.84 ,0.98 | 0.96 | 0.89 ,1.03 |
|  | Breast | 0.82 | 0.79 ,0.85 | 0.84 | 0.82 ,0.87 | 0.84 | 0.82 ,0.87 | 0.88 | 0.85 ,0.91 |
|  | Haematology | 0.39 | 0.37 ,0.42 | 0.40 | 0.38 ,0.42 | 0.42 | 0.39 ,0.44 | 0.44 | 0.42 ,0.47 |
|  | Head & Neck | 0.90 | 0.84 ,0.96 | 0.97 | 0.93 ,1.02 | 0.96 | 0.91 ,1.01 | 0.98 | 0.93 ,1.02 |
|  | Kidney | 0.92 | 0.86 ,0.98 | 0.99 | 0.94 ,1.03 | 1.00 | 0.95 ,1.05 | 0.97 | 0.93 ,1.02 |
|  | Liver | 0.79 | 0.72 ,0.87 | 0.81 | 0.75 ,0.87 | 0.82 | 0.76 ,0.87 | 0.88 | 0.83 ,0.93 |
|  | Lung | 0.82 | 0.79 ,0.85 | 0.82 | 0.80 ,0.84 | 0.81 | 0.79 ,0.84 | 0.83 | 0.81 ,0.86 |
|  | Oesophagus | 0.86 | 0.81 ,0.91 | 0.87 | 0.83 ,0.91 | 0.90 | 0.86 ,0.95 | 0.95 | 0.91 ,0.99 |
|  | Ovarian | 0.87 | 0.83 ,0.92 | 0.87 | 0.84 ,0.91 | 0.90 | 0.87 ,0.95 | 0.94 | 0.90 ,0.98 |
|  | Pancreas | 0.94 | 0.88 ,0.99 | 0.95 | 0.91 ,0.99 | 0.95 | 0.91 ,1.00 | 0.98 | 0.94 ,1.02 |
|  | Prostate | 0.96 | 0.86 ,1.07 | 0.99 | 0.91 ,1.07 | 1.00 | 0.92 ,1.09 | 0.94 | 0.86 ,1.03 |
|  | Stomach | 0.90 | 0.85 ,0.96 | 0.93 | 0.89 ,0.98 | 0.91 | 0.87 ,0.96 | 0.97 | 0.93 ,1.02 |
|  | Other | 0.86 | 0.83 ,0.89 | 0.88 | 0.86 ,0.90 | 0.88 | 0.86 ,0.91 | 0.92 | 0.90 ,0.95 |
| Marital status | Married | 1.00 | -- | 1.00 | -- | 1.00 | -- | 1.00 | -- |
|  | Divorced | 0.86 | 0.85 ,0.88 | 0.86 | 0.85 ,0.87 | 0.86 | 0.85 ,0.87 | 0.88 | 0.87 ,0.89 |
|  | Single | 0.75 | 0.74 ,0.76 | 0.75 | 0.74 ,0.76 | 0.74 | 0.74 ,0.75 | 0.76 | 0.75 ,0.77 |
|  | Widowed | 0.84 | 0.83 ,0.85 | 0.84 | 0.83 ,0.84 | 0.83 | 0.82 ,0.84 | 0.86 | 0.85 ,0.86 |
|  | Not stated/unknown | 0.91 | 0.88 ,0.95 | 0.88 | 0.84 ,0.92 | 0.82 | 0.78 ,0.86 | 0.83 | 0.80 ,0.86 |
| SES | 1 Most deprived | 1.00 | -- | 1.00 | -- | 1.00 | -- | 1.00 | -- |
|  | 2 | 1.03 | 1.01 ,1.04 | 1.03 | 1.01 ,1.04 | 1.03 | 1.02 ,1.04 | 1.02 | 1.01 ,1.04 |
|  | 3 | 1.04 | 1.02 ,1.06 | 1.04 | 1.03 ,1.06 | 1.06 | 1.05 ,1.08 | 1.06 | 1.05 ,1.07 |
|  | 4 | 1.05 | 1.03 ,1.07 | 1.06 | 1.04 ,1.07 | 1.10 | 1.09 ,1.12 | 1.09 | 1.08 ,1.10 |
|  | 5 Least deprived | 1.06 | 1.04 ,1.08 | 1.07 | 1.05 ,1.08 | 1.12 | 1.11 ,1.13 | 1.11 | 1.10 ,1.12 |
| Region | North West | 1.00 | -- | 1.00 | -- | 1.00 | -- | 1.00 | -- |
|  | East England | 0.88 | 0.86 ,0.90 | 0.92 | 0.90 ,0.94 | 0.93 | 0.91 ,0.94 | 0.95 | 0.94 ,0.96 |
|  | East Midlands | 0.80 | 0.78 ,0.82 | 0.86 | 0.84 ,0.87 | 0.86 | 0.84 ,0.87 | 0.88 | 0.86 ,0.89 |
|  | London | 0.81 | 0.79 ,0.83 | 0.82 | 0.81 ,0.84 | 0.87 | 0.86 ,0.89 | 0.89 | 0.87 ,0.90 |
|  | North East | 0.85 | 0.83 ,0.88 | 0.91 | 0.89 ,0.93 | 0.91 | 0.89 ,0.92 | 0.88 | 0.87 ,0.90 |
|  | South Central | 0.90 | 0.87 ,0.92 | 0.98 | 0.95 ,1.00 | 0.99 | 0.97 ,1.00 | 0.97 | 0.95 ,0.98 |
|  | South East Coast | 1.03 | 1.01 ,1.06 | 1.02 | 1.00 ,1.05 | 1.05 | 1.04 ,1.07 | 1.06 | 1.05 ,1.08 |
|  | South West | 0.94 | 0.92 ,0.97 | 0.92 | 0.90 ,0.94 | 0.93 | 0.92 ,0.95 | 0.98 | 0.97 ,0.99 |
|  | West Midlands | 0.97 | 0.95 ,0.99 | 0.95 | 0.93 ,0.96 | 0.92 | 0.91 ,0.93 | 0.95 | 0.94 ,0.96 |
|  | Yorkshire & Humber | 0.95 | 0.93 ,0.98 | 0.93 | 0.91 ,0.95 | 0.97 | 0.96 ,0.98 | 0.96 | 0.95 ,0.97 |

PRs were estimated from the log-binomial regression models. The clustering effect within the LSOA geographical units was adjusted using the general estimating equation (GEE) method. In additional to variables listed in the table and the interaction term of age and cancer, models were also adjusted for the calendar year of death. A PR greater than 1 indicates higher probability of death at home/hospice than the reference category. The overall p value for association of individual factors with place of death was smaller than 0.001 in all models.
